# Reference Platform for ADAS Camera System Evaluation

**DOI:** 10.3390/s25061690

**Published:** 2025-03-08

**Authors:** András Rövid, Zsolt Vincze, Tamás Pálinkás, Mihály Kocsis, Viktor Serrano, Zsolt Szalay

**Affiliations:** 1 Department of Automotive Technologies, Budapest University of Technology and Economics, Stoczek Str. 6, 1111 Budapest, Hungary; vincze.zsolt@kjk.bme.hu; 2 Robert Bosch Kft., Gyömrői Str. 104, 1103 Budapest, Hungary; tamas.palinkas2@hu.bosch.com (T.P.); mihaly.kocsis2@hu.bosch.com (M.K.); viktor.serrano@hu.bosch.com (V.S.)

**Keywords:** ADAS, camera-based systems, reference measurement, object detection, automotive safety, sensor validation

## Abstract

Advanced driving assistance systems (ADASs) are critical for automotive safety. They rely on various sensors (especially with an increasing reliance on visual sensors to meet evolving safety standards) to capture relevant environmental data. The validation of ADAS systems is crucial to ensure their reliability and performance in real-world driving scenarios; however, this requires reference data. This paper focuses on the development of a reference sensor system that can provide reference data and does support the validation of visual sensors for ADAS systems. The system is validated in various relevant scenarios at an automotive proving ground.

## 1. Introduction

Advanced driving assistance systems (ADASs) are an essential part of today’s automobile industry [1]. These systems use different type of sensors to gather the necessary environmental information  [2]. Validating ADAS systems is a critical aspect of ensuring their safety, reliability, and effectiveness in real-world diverse driving scenarios [3]. The number of systems that are using visual sensors are constantly expanding, because camera-based systems are required for the automotive manufacturers to fulfill the latest safety regulations [4,5].

During the development, it is necessary to evaluate the performance of these sensors and for that, a reference measurement system (having an increased precision compared to the evaluated system) shall be adopted that can be used as reference (ground truth). There is no universal solution for this problem, and developing such a system is a challenging task.

The development of the presented reference system was part of a project in which a specific use case related to the performance of the front sensing camera was analyzed, since the front sensing camera cannot be installed in the optimal position behind the windscreen in several vehicle types. The goal of this project was to perform an investigation on the effect of different camera mounting positions and tolerances on the ADAS performance.

The paper introduces the process of developing a reference sensor system by focusing on the key aspects of the development that need to be considered, the use cases, the advantages, disadvantages, and the limitations.

The reference data from the reference sensor system can also be transformed into virtual scenarios, which enables the rapid assessment of the impact of changes applied on the vehicle under test [6].

In [7], a plug-and play ground-truth reference device is presented, and also the challenges in calibration of the reference sensor system, as well as the data postprocessing pipeline, are explained in detail. In [8], the framework to estimate the distance between a vehicle equipped with sensors and different road objects is explained.

In [9], the authors proposed a reference system where an UAV equipped with a 4K camera flies above a test track to evaluate an automotive perception system. Computer vision methods are employed to accurately determine the positions of objects around the car, utilizing ArUco markers and a deep convolutional neural network. In [10], a modular system architecture is presented for fusing data from environment sensors in advanced driver-assistance systems (ADAS). The architecture enables various applications to access the fused sensor data by processing it according to the specific requirements of different application groups. This flexible approach aims to enhance the functionality and effectiveness of ADAS by providing tailored data integration solutions. The authors in [11] present an approach that relies on the complementary use of three data sources, namely, a highly precise 3D map with semantic information, a high-density range finder sensor, and a GNSS-RTK/INS localization unit. In string relation to reference measurement systems, the authors in [12] introduce a baseline reference framework for sensor context and derive multidimensional schemata representing different modeling and analysis scenarios.

The paper is organized as follows: Section 2 gives an overview on the applied methodology, Section 3 deals with requirements relevant for the proposed reference measurement system, and Section 4 introduces the high-level architecture of the measurement platform including the software, as well as hardware elements. Section 5 provides details on the proposed and applied algorithms, while Section 6 focuses on the validation of the proposed system, through various scenarios, which were performed at the ZalaZONE automotive proving ground. Finally, a discussion on the pros and cons is presented, followed by a succinct report of the conclusions drawn from the project’s findings.

## 2. Methodology

**Figure 1:**
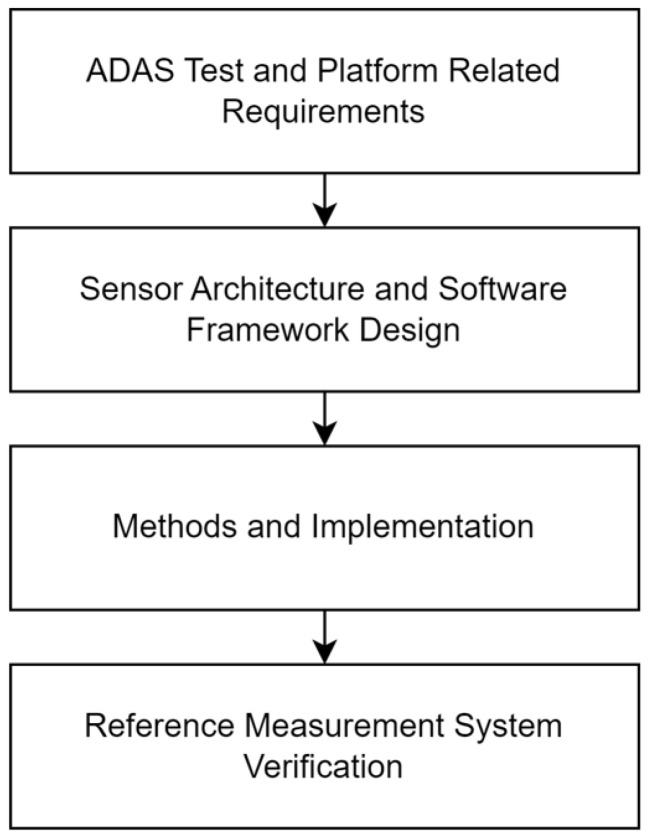
Research procedure diagram of the work.

This section is devoted to overview the main stages (see Figure 1) of the applied methodology related to the elaboration of the proposed reference measurement platform. Firstly, the requirements for the measurement platform have to be defined, which, in our case, included the collection of hardware- and software-related requirements. A portion of the requirements is strongly related to the EURO NCAP test specification protocol, while a part of the requirements came from stakeholders, mainly related to the minimization of test preparation and evaluation time. In Section 3, the requirements are discussed in more detail.

After the requirements are defined, the next step relates to the planning stage. In the case of the proposed reference measurement platform, this stage is covered by planning where the sensors should be placed and what types they have to be in order to obtain the same FOV as that of the ADAS camera under test (see Section 4.1). On top of that, each sensor has to be time-synchronized with the master clock and properly calibrated.

From the software architecture design point of view the main objective was to provide a framework that can obtain time-synchronized raw data frames from both the reference sensors and that of the sensor under test, extract the targets from the raw reference frames (camera images and LiDAR pointclouds), associate the targets with the objects provided by the sensor under test, and perform evaluation (see Section 4.2).

The third stage relates to the implementation, during which the system is implemented from both hardware- and software-related aspects (see Section 5). First, camera and LiDAR sensors are mounted on top of the vehicle such that their FOV covers the whole FOV of the camera under test. In addition, the hardware components necessary to interconnect the sensors are deployed and the components provide a reference time source for synchronization (see Section 5.4). After the configuration of hardware components, the sensors are calibrated (see Section 5.3). In addition to hardware deployment and configuration, the methods for detecting and associating objects with the objects detected by the camera under test are elaborated and implemented.

Finally, in the verification stage, the whole reference measurement platform is verified, where a differential GNSS sensor was used as reference in both the EGO vehicle and the target vehicle to verify the accuracy of the system in different scenarios (see Section 6).

## 3. Reference System Requirements

During the configuration of the reference system architecture, the first step is to define the requirements. Deep knowledge about the properties, the features, and the limitations of the examined system is inevitable. It is also important to consider the environmental conditions, the use cases, and, as shall be decided, which features are to be verified with the reference system.

In this project, the system under test is a monocular-camera-based advanced driver assistance system. This multi-purpose smart camera provides the most common driver assistance features like lane keeping assistant, adaptive cruise control, autonomous emergency braking, road sign recognition, and adaptive headlight control with only a single video sensor. The primary goal of the reference system is to create ground-truth data for the object detection algorithm of the system. This ADAS is used on public roads with continuously changing environmental and road conditions; hence, the most important criteria is to develop a reference system in which usage is not limited to test tracks but can also be used on public roads where the more complex traffic situations can challenge the video sensor. This means that only the vehicle under test (VUT) could be equipped with sensors, and the environment cannot be prepared for the reference system. The reference system shall detect and classify the objects and other participants, provide the object attributes, and automatically associate them with the detected objects of the ADAS.

A monocular-camera-based ADAS has several advantages and disadvantages. These systems estimate the distance of the detected objects based on different techniques and assumptions, which means the accuracy of monocular systems is generally worse compared to a stereo camera or a radar-based system. The distance error tends to be higher as the distance increases; therefore, the scope of the reference system is not to cover the whole range of the detection distance of the ADAS but to provide the object attributes reliably within a shorter range, which is particularly crucial for optimizing the performance of the assistance features.

The type and the number of the reference system sensors are based on several criteria that need to be considered. In order to fulfill the latest safety regulations like Euro NCAP [13], the ADAS has to have a wide field of view (FOV). The reference system shall cover the full range of the FOV of the sensor under test and also has to be prepared for the future generation of ADASs with even higher FOV. The camera-based ADAS runs several detection algorithms, for instance, the road sign, light object, and lane and road boundary detection, which can only be validated with a camera-based system; therefore, the reference system has to include camera sensors as well. For the reliable and precise distance measurement, a LiDAR is also included in the system architecture. It is also beneficial if the reference system includes sensors that are using different sensing techniques, because they can compensate for the disadvantages of each sensor type.

During the selection of the sensors, another key aspect is the synchronization. The clocks of all sensors (cameras, LiDAR), as well as the clock of the sensor under test and that of the central computer, have to be synchronized properly in order to associate the corresponding camera and LiDAR frames. For this purpose, a precision time protocol (PTP) time master is used as the time source to synchronize the clocks.

## 4. Reference Platform Architecture Design

This section provides the description together with the high-level hardware and software architectures of the proposed reference measurement platform.

### 4.1. Sensor Architecture Design

**Figure 2:**
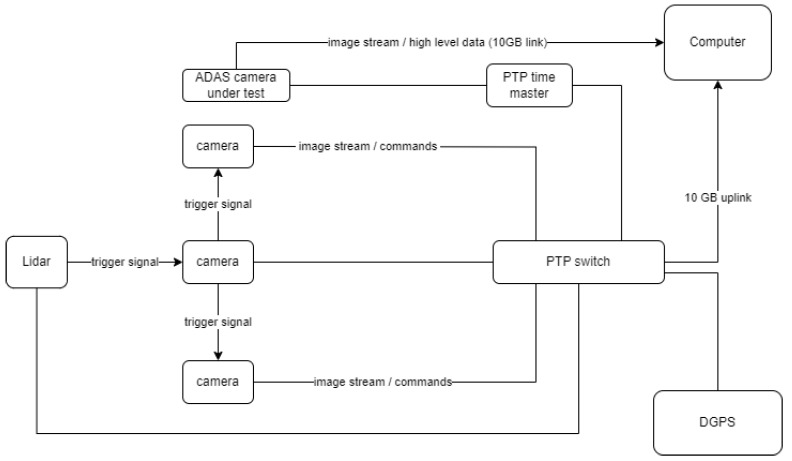
High level architecture of the reference measurement system.

The reference measurement system is composed of front facing cameras (together covering the FOV of the sensor under test), a rotational LiDAR sensor, a PTP time master, a GNSS system with RTK correction support, and a computer (see Figure 2). The clocks of these devices are synchronized using the precision time protocol (PTP). In addition, the cameras are triggered by a square wave signal where the timing of the rising edge corresponds to the time when the LiDAR beams begin scanning the middle of the FOV covered by the front facing cameras. In ref. [14], the concepts and tools for analyzing and setting the timestamp of sensors are discussed, by focusing on two classes, namely, when the global time generator allows the creation of a relationship between the measurement and the acquisition timestamps and when a synchronization signal is periodically sent to the sensors that timestamp their data relative to this reference signal.

Our proposed system touches both classes: the trigger signal generated by the LiDAR is used to trigger the multi-camera system, and each device (including the sensors and the computer) receives the time-synchronization messages from a time master by relaying on the PTP (see Figure 2).

### 4.2. High-Level Architecture of the Evaluation Framework

**Figure 3:**
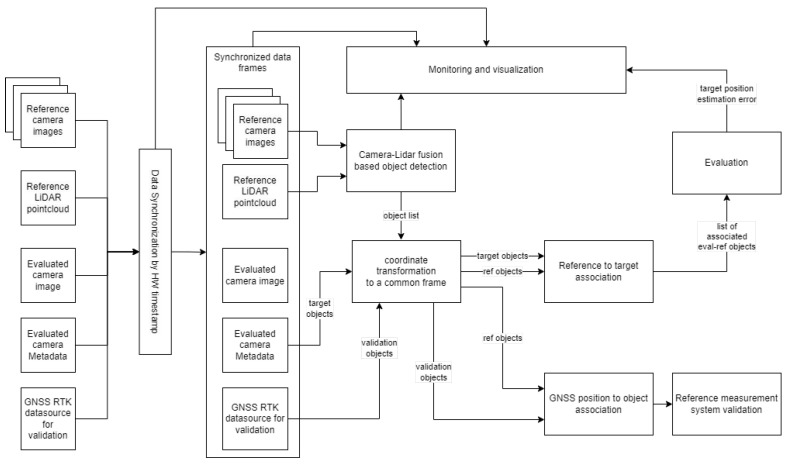
High level architecture of the evaluation pipeline of the detector under test.

In Figure 3, the high-level architecture of the proposed evaluation pipeline of the sensor under test is depicted. The reference data (camera images, LiDAR pointcloud, GNSS position and heading) and the data (images, metadata) of the sensor to be evaluated are fed into the evaluation framework, where based on hardware timestamps (the time when the camera image/LiDAR pointcloud was acquired by the sensor), the assignment of corresponding frames is performed. From such obtained synchronized data, the objects are extracted by applying a 3D object detector (see Section 5.1). The detector relies on both camera image and LiDAR pointcloud to estimate the position of the target. The extracted target position and the location of the same target estimated by the sensor under test, as well as its UTM coordinates, are all transformed into a common coordinate system (see Section 6.1). As the system can handle multiple targets at the same time, it is crucial to perform reference to target assignment, which will allow us to evaluate the accuracy of the sensor under test. The association is performed by using the global nearest neighbor association algorithm. The GNSS-based RTK-corrected position data serve as reference locations to validate the detector used in the proposed reference measurement system.

## 5. Methods and System Integration

The methods covered in this section are related to the components of the system (see Figure 3) performing object detection and association, as well as the methods related to the calibration of the proposed measurement platform.

In the next section, let us point out in more detail the object detector used by the proposed reference system, which was validated in multiple scenarios in a controlled environment.

### 5.1. Camera–LiDAR Fusion-Based Detector

The detector used by the system operates on multi-camera images and LiDAR pointclouds, meaning that both the higher spatial resolution of camera images (compared to LiDAR pointclouds) and the higher depth resolution of LiDARs can be utilized jointly to achieve increased detection performance. The detection of objects is performed in the pixel space, whereas the depth estimation is based on LiDAR measurements. The detector builds upon the approach introduced in [15]. The detector used by the proposed system searches for the nearest LiDAR point to the camera center for each target within the joint field of view of the multi-camera system. Here, the camera system is equipped with hardware synchronization, as mentioned in the previous section.

Let $\mathrm{PS}=(P_{1},P_{2},...)$ and $I=(I_{1},I_{2},...)$ stand for point-cloud and image time series, respectively. Obviously, first, pairs $\left(P_{i},I_{j}\right)$, being closest in time, are selected for processing based on their timestamps; that is, $|timestamp\left(P_{i}\right)-timestamp\left(I_{j}\right)|$ is minimal. The processing phase can be decomposed into two main steps: Firstly, the objects are detected in the pixel space, and their bounding boxes are estimated. Secondly, based on the bounding box estimates and the camera–LiDAR calibration, for each object, the LiDAR point closest to the camera center is determined.

To detect objects in the camera image space, the YOLOv5 [16] neural network architecture was used. Further promising alternatives are the YOLO-NL model [17], fuzzy-attention-based YOLO model [18], or YOLOv8-based models published, for instance, in [19].

**Figure 4:**
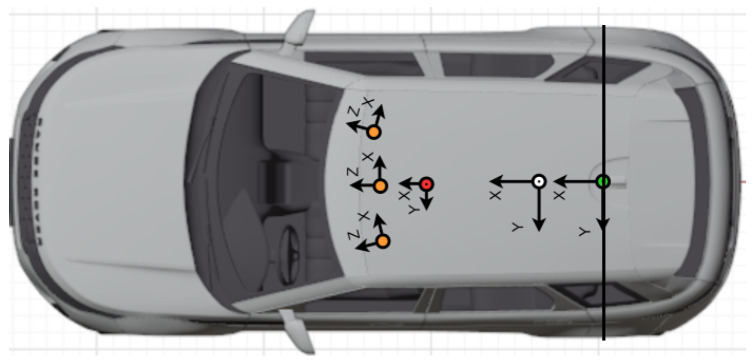
The coordinate systems defined for the reference measurement vehicle: $S_{L}$: LiDAR frame (red), $S_{cam}^{\left(j\right)}$: *j*th camera frame (orange), $S_{imu}$: IMU frame (white), $S_{ref}$: attached to the middle of the rear axle (green). The target object is localized with respect to $S_{ref}$.

Let $C_{j}$ denote the camera center and $X_{k,j}$ the frustum defined by $C_{j}$ and the bounding box $B_{k,j}$ of the *k*th object in the image space of the *j*th camera. The coordinate systems considered in the case of the EGO vehicle are depicted in Figure 4. The multi-camera–LiDAR object detector can be followed in Algorithm 1.

The detector detailed in Algorithm 1 represents a promising and simple approach for supporting reference data collection online. Even though this method does not provide the heading of the target, a single LiDAR ray intersecting with the vehicle is sufficient for position estimation. Although at longer distances, the error associated with this estimate strongly depends on the density of points representing the target vehicle, it remains within the ground-truth bounding box of the vehicle.

Additional detectors can also be incorporated into the proposed measurement framework. Promising camera–LiDAR-fusion-based detectors can be followed, for instance, in [20,21,22], LiDAR-only-based alternatives for real-time object detection are discussed in [23,24,25]. Further distance measurement systems including vision-based techniques, millimeter wave radars, infrared ranging, and LiDAR, as well as their designs, are discussed in [26]. A processing pointcloud time-series can provide additional time encoded information, which is beneficial for 3D object detection tasks, as well. Such methods are introduced in [27].
**Algorithm 1** Detection of objects based on multi-camera and LiDAR data**Require:** LiDAR pointcloud P**Require:** Camera images $I^{\left(1\right)}$, $I^{\left(2\right)}$, $I^{\left(3\right)}$ acquired by the 1st, 2nd, and 3rd camera, respectively. (In case of purely rotated cameras, a single panoramic image can be used, composed from $I^{\left(1\right)}$, $I^{\left(2\right)}$, $I^{\left(3\right)}$ covering the same FOV as the FOVs of the involved cameras jointly.)    **Initialize**: *object list*← []    **for each** camera *j* where j=1,2,3 **do**          Apply YOLOv5 model on $I^{\left(j\right)}$          Project each point $P_{i}\in C$ onto the camera image plane (considering undistorted          camera image):(1)$$p_{i,j}=K_{j}\left[R_{j}|t_{j}\right]P_{i},$$          where $P_{i}\in P^{3}$, $K_{j}$ stands for the camera matrix of the *j*th camera given by (2); the          rigid transformation from LiDAR to the *j*th camera is given by rotation $R_{j}\in SO(3)$ and          translation $t_{j}\in R^{3}$.(2)$$K_{j}=\left[\begin{array}{ccc} f_{x,j} & 0 & c_{x,j}\\ 0 & f_{y,j} & c_{y,j}\\ 0 & 0 & 1 \end{array}\right],$$          where $f_{x,j}$ and $f_{y,j}$ stand for the focal length in terms of pixel dimensions, while $c_{x,j}$          and $c_{y,j}$ represent the principal point coordinates in terms of pixel dimensions for the          *j*th camera.          **for all** object *k* extracted from camera image $I_{j}$ **do**                $P^{\prime }\leftarrow P\cap X_{k,j}$ ($P_{i}\in X_{k,j}⟺p_{i}\in B_{k,j}$)                $P^{*}=\arg\min_{P_{i}\in P^{\prime }}∥P_{i}-C_{j}∥$                $P^{\prime }=T_{L}P^{*}$, where $T_{L}$ stand for the rigid transformation from LiDAR to $S_{ref}$                Put $P^{\prime }$ into *object list*          **end for**    **end for**

### 5.2. Association and Tracking

In order to track the points $P_{j}^{\prime }$ (corresponding to the *j*th detected object), j=1…N, where *N* stands for the number of detected targets, the global nearest neighbor (GNN) multi-object tracker was used. To associate the reference points $P_{j}^{\prime }$ to existing tracks, the GNN assignment algorithm was utilized, which minimizes the cost in (3) to obtain the assignment matrix $A^{*}$.

(3) $$\begin{array}{c} A^{*}=\arg\min_{A}\sum_{i=1}^{N}\sum_{j=1}^{M}d_{ij}a_{ij},\\ s.t.\\ \sum_{i=1}^{N}a_{ij}=1,\forall j\;\mathrm{and}\;\sum_{j=1}^{M}a_{ij}=1,\forall i \end{array}$$

The cost matrix element $d_{ij}$ stands for the Mahalanobis distance of the *i*th track and the *j*th detection. The interacting multiple model filter is utilized to predict the future location of the target point $P_{j}^{\prime }$, where the applied motion models were the constant velocity, constant acceleration, and constant turn rate models [28,29,30].

Similarly, to associate the detections provided by the camera under test with the existing reference tracks, the minimization problem in (3) is solved again, given the cost matrix $D^{\prime }$, where element $d_{ij}^{\prime }$ corresponds to the Mahalanobis distance of the *i*th track and the *j*th detection provided by the camera under test. By having this assignment, the camera under test can be evaluated.

### 5.3. Calibration

Calibration of the camera intrinsics and the camera–LiDAR extrinsics is essential for accurate position estimation of the target. The camera intrinsics and extrinsics were estimated by the method published in [31].

For estimating the LiDAR–camera extrinsics, a checkerboard-based approach was applied, where the checkerboard corners $X_{i}$ in the LiDAR coordinate frame are estimated by segmenting the plane of the checkerboard in the pointcloud and fitting the checkerboard model to the segmented 3D points. The corners can then be obtained directly from the fitted model [32,33]. After the checkerboard corners are determined, the following cost function is to be minimized to obtain the rigid transformation from LiDAR to camera:

(4) $$\min_{R,t}\sum_{i=1}^{N}{∥X_{i}^{\prime }-\left(RX_{i}+t\right)∥}^{2},$$

where $X_{i}^{\prime }$ and $X_{i}$ are the checkerboard corner points in the camera and the LiDAR coordinate system, respectively. *R* and *t* stand for the rotation and translation from LiDAR to camera coordinate system. Since, in the proposed reference system, there are three cameras with slightly overlapping FOVs and one LiDAR, the above pose estimation is performed for each camera–LiDAR pair.

A thorough investigation on how the placement of the camera and LiDAR affects 3D object detection is detailed in [34].

### 5.4. The Integrated System Components

**Figure 5:**
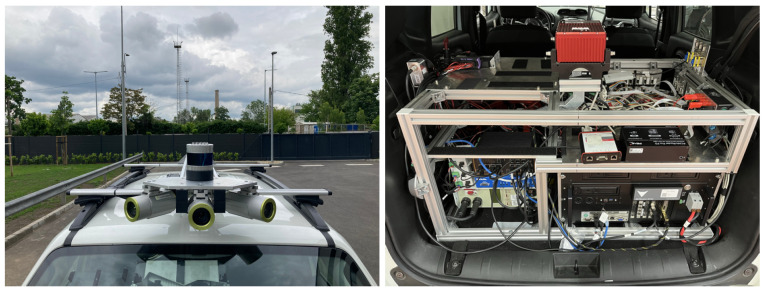
Setup of the reference measurement system.

In this section, the real platform (see Figure 5) and the measured latencies between the sensors and the proposed ROS-based framework (see Table 1) can be followed.

## 6. System Verification

The verification of a newly established system is crucial to ensure that the reference system fulfills the requirements. The following section describes the verification test measurement setup, the defined scenarios, and the results of the measurements.

### 6.1. Measurement Setup

**Figure 6:**
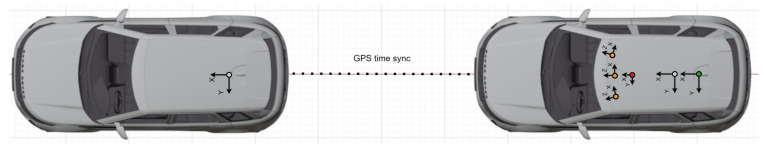
The coordinate systems of the target and EGO vehicle: LiDAR (red), camera (orange), GNSS/IMU (white), rear axle (green). The target is localized with respect to the rear axle coordinate system. The EGO and target clocks are synchronized by GPS time.

**Figure 7:**
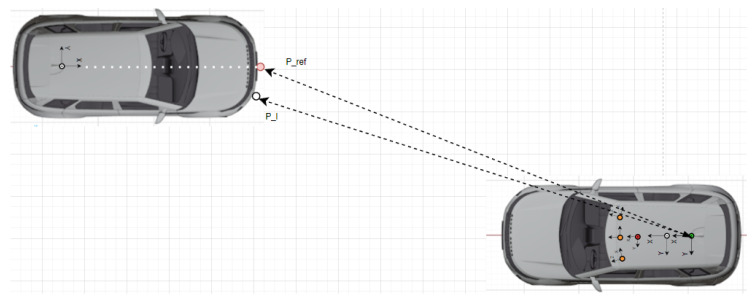
The reference point $P_{ref}$ (pink) and the LiDAR point $P_{l}$ (white) detected as the nearest available LiDAR point of the target with respect to the EGO reference frame $S_{ref}$. The coordinate systems of the LiDAR (red), cameras (orange), GNSS/IMU (white), and rear axle (green) are also illustrated.

**Figure 8:**
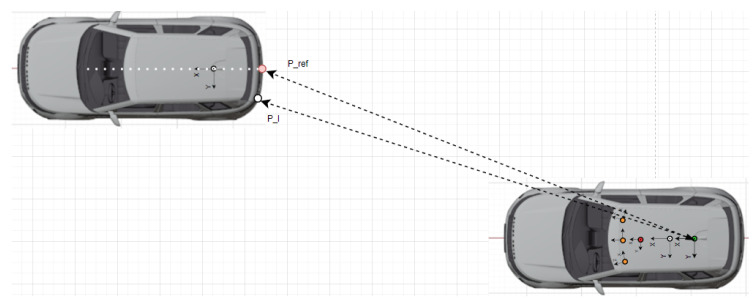
The reference point $P_{ref}$ (red) and the LiDAR point $P_{l}$ (white) detected as the nearest available LiDAR point of target with respect to the EGO reference frame $S_{ref}$. The coordinate systems of the LiDAR (red), cameras (orange), GNSS/IMU (white), and rear axle (green) are also illustrated.

Verification measurements were carried out with one target vehicle that is equipped with a GNSS/IMU RTK system, as depicted in Figure 6, Figure 7 and Figure 8. In order to validate the proposed measurement system by relying on the GNSS RTK reference position of the target expressed by homogeneous coordinates $P_{ref}={\left[X,Y,Z,1\right]}^{T}$, the measurements have to be transformed into a common coordinate system $S_{ref}$, which, in our case, is the frame attached to the rear axle of the EGO vehicle (see Figure 6).

Let us denote the rigid transformation from the UTM to the EGO vehicle’s IMU frame $S_{imu}$ by $T_{imu}\in SE(3)$ and let $T_{ref}\in SE(3)$ denote the rigid transformation from $S_{imu}$ to $S_{ref}$. The detected closest point $P_{L}=\left[X_{L},Y_{L},Z_{L},1\right]$ of the target is obtained in the LiDAR coordinate system $S_{L}$ and is rigidly transformed into $S_{ref}$ by $T_{L}\in SE(3)$. Thus, the position error vector $\epsilon ={\left[e_{x},e_{y},e_{z},0\right]}^{T}$ is obtained as follows:

(5) $$\epsilon =T_{L}P_{L}-T_{ref}T_{imu}P_{ref}$$

The latency of the proposed reference measurement system can be followed in Table 1.

### 6.2. Defined Scenarios and Results

The detailed designs of all the tests are documented in the test catalog, which is required for the verification. The reference system is primarily designed to validate camera-based ADASs; therefore, the verification of the reference system is also focusing on basic static and dynamic scenarios which are typical for an ADAS feature like adaptive cruise control (ACC) and autonomous emergency braking (AEB).

To ensure the repeatability of the tests, the EURO NCAP test specification protocol [35] was used as a base to define the required weather conditions in order to minimize their effect on the results. This protocol focuses on evaluating the performance of the ADAS features. There are several parameters that are not relevant for the performance of the object detection; therefore, the following parameters were considered:

- The ambient temperature shall be between 5 °C and 40 °C.
- The tests shall be conducted in dry conditions
- Homogeneous, natural ambient illumination is required, avoiding any excess shadows in the test area.
- During the test execution, driving towards or away from direct sunlight shall be avoided.

The defined scenarios and the results are detailed in the next section. The arrow on the pictures of the scenarios represent the movement direction of the vehicles. The diagram on the left shows the distance error of the reference system compared to the GNSS RTK system, while the diagram on the right represents the relative longitudinal velocity between the VUT and the target vehicle.

#### 6.2.1. Scenario-1: Car-to-Car Rear Stationary (CCRs)

**Figure 9:**
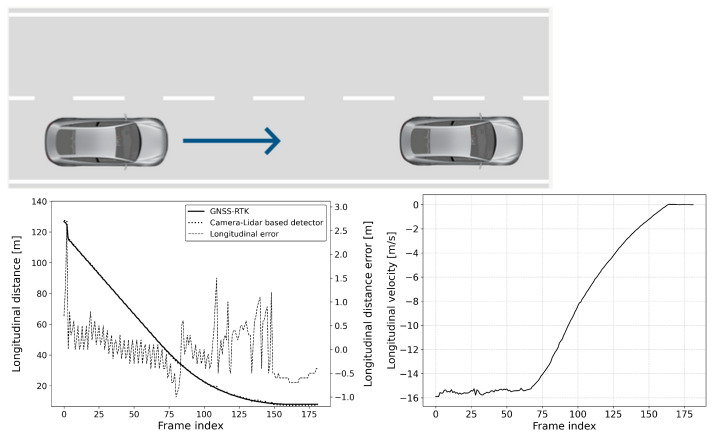
CCRs vVUT: 60 km/h; Longitudinal distance and its error compared to the GNSS-RTK-based reference (**left**); Relative longitudinal velocity of the target with respect to VUT across the acquired data frames (**right**).

**Figure 10:**
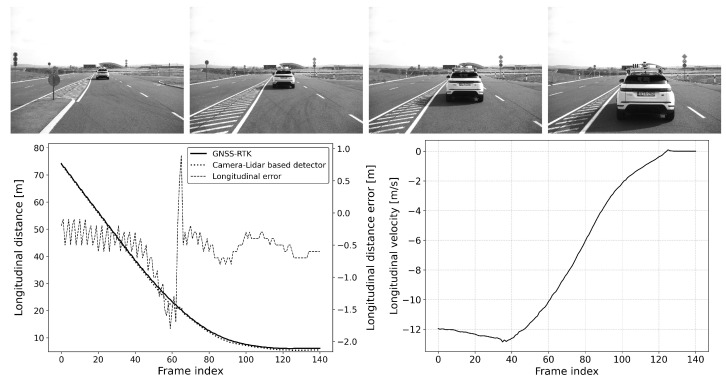
CCRs vVUT: 40 km/h; Longitudinal distance and its error compared to the GNSS-RTK-based reference (**left**); Relative longitudinal velocity of the target with respect to VUT across the acquired data frames (**right**).

This scenario is common for an automatic emergency braking (AEB) function and is also part of most standard safety regulations (see Figure 9 and Figure 10). In this scenario, the vehicle under test (VUT) approaches a stationary target vehicle from a far range, initially beyond the detection distance. This setup allows for the precise determination of the maximum detection distance and measurement error, given the fixed position of the target vehicle. The VUT travels at speeds ranging between 40 and 80 km/h with 10 km/h speed steps, providing a consistent and controlled environment to evaluate the performance of the AEB system. This evaluation is crucial for ensuring that the AEB system can reliably detect obstacles and activate braking in time to prevent collisions, thereby enhancing vehicle safety and compliance with regulatory standards.

#### 6.2.2. Scenario-2: Approaching a Moving Vehicle

**Figure 11:**
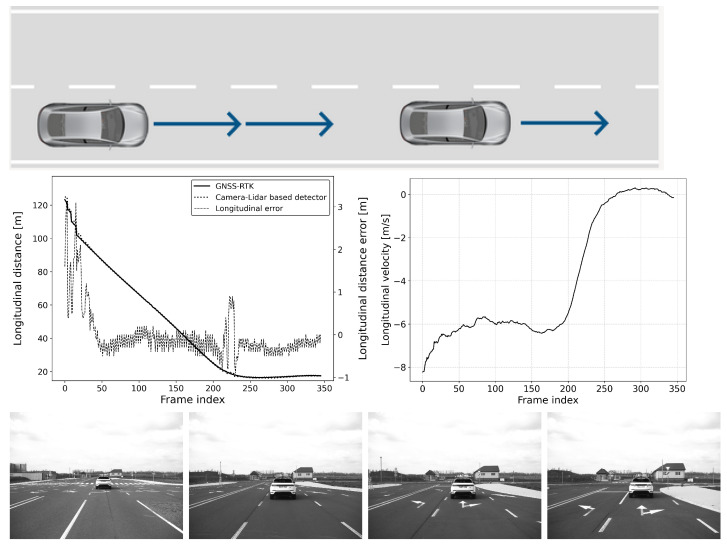
Approach: vVUT: 60 km/h, vTarget: 40 km/h; Longitudinal distance and its error compared to the GNSS-RTK-based reference (**left**); Relative longitudinal velocity of the target with respect to VUT across the acquired data frames (**right**).

**Figure 12:**
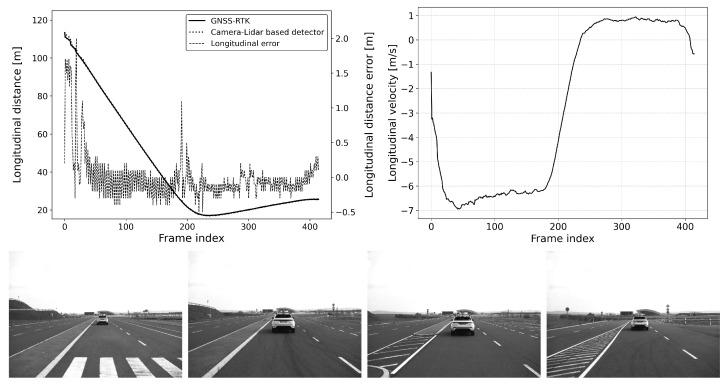
Approach: vVUT: 40 km/h, vTarget: 20 km/h; Longitudinal distance and its error compared to the GNSS-RTK-based reference (**left**); Relative longitudinal velocity of the target with respect to VUT across the acquired data frames (**right**).

This situation is common for an adaptive cruise control (ACC) system (see Figure 11 and Figure 12). In this scenario, the vehicle under test (VUT) approaches a target vehicle moving in the same direction with constant velocity. This setup allows for a comprehensive comparison of detection performance between static and dynamic scenes. By varying the speeds of both vehicles, the effect of different relative velocities on the ACC system’s performance can be thoroughly investigated.

The VUT travels at speeds between 50 and 80 km/h, while the target vehicle’s speed ranges from 20 to 40 km/h. All combinations of the VUT and the target vehicle’s speed range with 10 km/h steps were tested. This variation in speed provides valuable data on how well the ACC system can adapt to changing traffic conditions, maintain a safe following distance, and ensure smooth acceleration and deceleration. Understanding these dynamics is essential for optimizing ACC system algorithms and ensuring their reliability and effectiveness in real-world driving scenarios.

#### 6.2.3. Scenario-3: Car-to-Car Front Turn-Across-Path (CCFtap)

**Figure 13:**
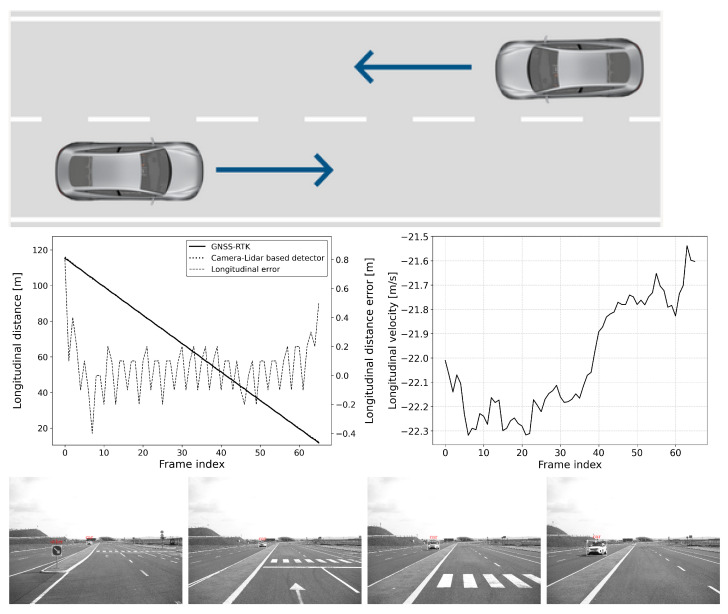
CCFTap: vVUT: 40 km/h, vTarget: 40 km/h; Longitudinal distance and its error compared to the GNSS-RTK-based reference (**left**); Relative longitudinal velocity of the target with respect to EGO vehicle across the acquired data frames (**right**).

**Figure 14:**
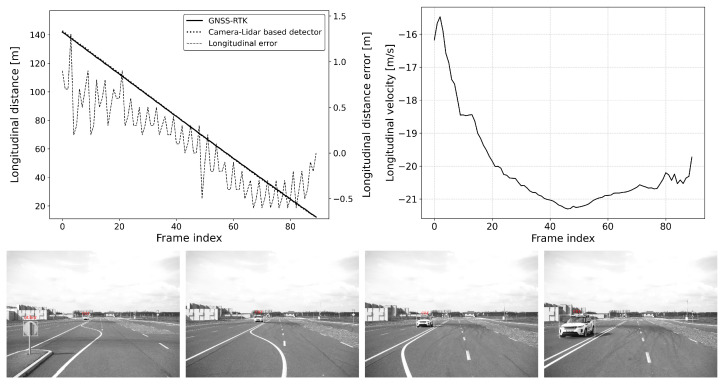
CCFTap: vVUT: 40 km/h, vTarget: 30 km/h; Longitudinal distance and its error compared to the GNSS-RTK-based reference (**left**); Relative longitudinal velocity of the target with respect to the EGO vehicle across the acquired data frames (**right**).

**Figure 15:**
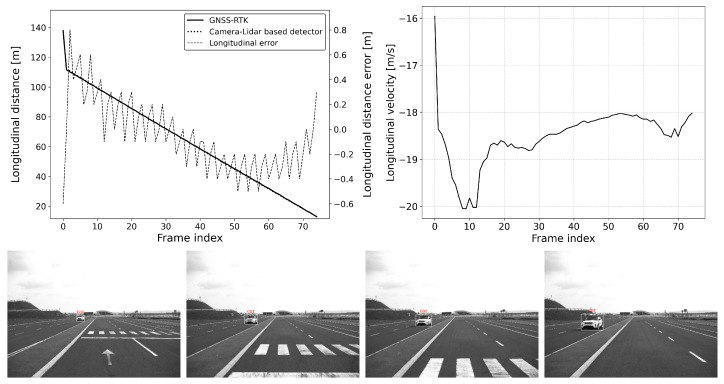
CCFTap: vVUT: 30 km/h, vTarget: 20 km/h; Longitudinal distance and its error compared to the GNSS-RTK-based reference (**left**); Relative longitudinal velocity of the target with respect to the EGO vehicle across the acquired data frames (**right**).

This is also a typical scenario for an automatic emergency braking (AEB) function, where the system must react to an oncoming target object (see Figure 13, Figure 14 and Figure 15). In this scenario, the vehicle under test (VUT) approaches a target vehicle moving towards the VUT in the adjacent lane. This setup provides an opportunity to verify the detection distance and accuracy of the AEB system under dynamic conditions, especially when there is a high relative velocity between the VUT and the target vehicle. The VUT and the target vehicle both travel at speeds between 20 and 40 km/h, and as in the other scenarios, all combinations of the VUT and target vehicle’s speed range with 10 km/h steps were tested.

By examining the AEB system in such a dynamic scenario, engineers can assess its ability to accurately detect and respond to fast-approaching vehicles, which is critical for preventing head-on collision. The test will measure how quickly and accurately the AEB system can identify the threat, calculate the appropriate response, and engage the braking system to mitigate or avoid an impact. This scenario is particularly important for ensuring the reliability and effectiveness of AEB systems in real-world driving conditions where vehicles often encounter oncoming traffic at varying speeds. Such rigorous testing helps in refining the system’s algorithms to enhance its performance, thereby contributing to vehicle safety and compliance with stringent automotive safety standards.

#### 6.2.4. Observations

The system demonstrates an average error of less than 1 m throughout the entire detection range, making it suitable for validating camera-based advanced driver assistance systems (ADASs). This level of accuracy ensures that the system can reliably support functions such as lane-keeping, collision avoidance, and adaptive cruise control by providing the precise positional data of surrounding objects.

However, transient errors can be observed whenever the target transitions to a new motion state, such as a change in speed or direction. These transient errors are temporary deviations in accuracy that occur during the adjustment period following the target’s motion change. For example, such transitions can be clearly followed in Figure 11 and Figure 12, specifically at frames 225 and 190, respectively. Due to such transitions, a slight increase in error can be observed; however, these transients settle down quickly, and the absolute error of the location estimate returns to the interval [−0.5,0.5] meters.

**Figure 16:**
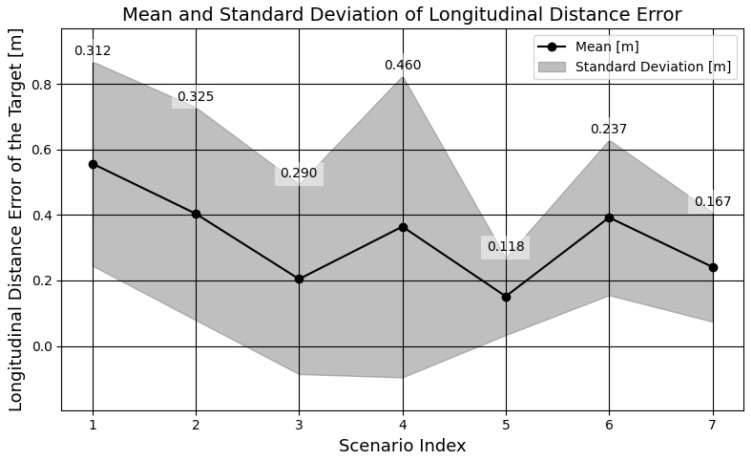
Mean and standard deviation of longitudinal distance error across scenarios.

**Figure 17:**
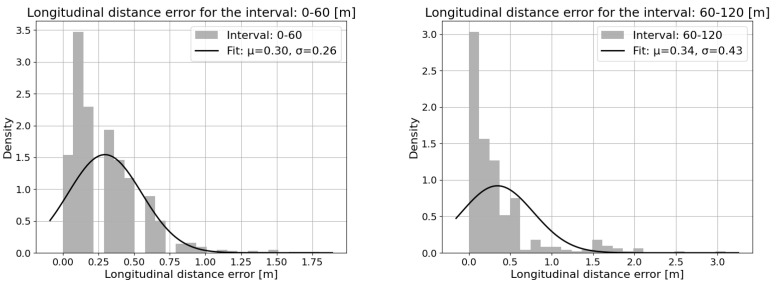
Longitudinal distance measurement error distribution for the range (EGO–target distance) [0–60] m (**left**) and [60–120] m (**right**).

Figure 16 contains the mean and standard deviation of longitudinal distance error across the different scenarios, which clearly shows that the distance error is consistent and within a 1 m range. Figure 17 shows the longitudinal distance measurement error distribution for the detection range distributed into close (0–60 m) and long range (60–120 m), as the distance error increases with the range.

## 7. Discussion

Let us point out the advantages of the proposed solution over the most commonly used reference data generation approaches. The following table (see Table 2) contains a short comparison about the most commonly used validation methods in the automotive industry.

One of these is the differential GPS (dGPS), which, on one hand, offers high accuracy; the weather conditions are not limiting its usage, but, on the other hand, the number of targets, or objects that can be investigated is strongly limited due to the high cost of these devices. In addition, setting up a complex dGPS reference system is time consuming.

Some reference systems use drones, which are easy to set up and can be a good solution to handle complex scenarios as well. However, the usage of these systems in public environments is strictly regulated; nevertheless, the speed and range of the drones also limits their usability, as described in [9].

The introduced reference system unlocks significant potential for the development of ADASs. There are several traffic situations that are hard to reproduce in the test track environment; therefore, it is a huge advantage that the proposed reference system can be used on public roads as well. The proposed system has a much higher detection distance than the one that is described in [8].

Although the proposed system widens the testing possibilities, different weather conditions are limiting its usage. In [36], a comprehensive methodology for exposure time optimization under various lighting conditions is presented.

Heavy rain can influence the performance, especially in the case of camera-based detectors, and the performance of the camera sensor is also influenced by the low or blinding lightning conditions. Glare can also have a negative impact on the performance of the proposed system; however, it can significantly be reduced by placing a polarizing filter in front of the camera lens. In the case of using neural models for object detection, the detection performance under various weather conditions might be improved by training the models on training data in which scenarios acquired under different weather conditions are equally represented.

Snowflakes, fog, and condensed moisture can interfere with the pointcloud of the LiDAR and highly affect the performance of the sensor. The snowflakes, for instance, cause impulse-like noise in the pointcloud provided by the LiDAR sensor. Such types of noise can significantly be reduced by applying median filtering on the pointcloud.

## 8. Conclusions

A large amount of valuable data was recorded that greatly contributed to the development of ADASs. The system greatly fulfills its purpose and provided unique data for the development. However, there is always room for improvement.

In order to precisely evaluate the performance of a sensor, a great amount of measurements and data have to be generated. The detailed testing of a sensor under the most severe weather conditions and challenging situations requires a lot of effort and might be impossible to create such conditions and test all the corner cases. The reference system and the evaluation toolchain is prepared to process also virtual and augmented testing methods with which the number of tests could be greatly increased. The implementation of such testing methods would significantly reduce the cost of developing such ADASs.

Although we have discussed the verification of the system in this article, a more detailed validation process has to be developed. The validation concept shall include various weather conditions, traffic situations, and the system shall be tested in different static and dynamic scenarios as well. With this approach, the effect on the performance of different object types, situations, and environmental conditions can be mapped.

The system has the potential to extend the usage for other sensor types. The most common ADASs use cameras, ultrasonic, and radar sensors. Because of the high cost, LiDAR sensors are not so frequently used. The requirements for these sensors depend on the ADAS features that need to be realized; therefore, the front-facing radar and LiDAR sensors have similar requirements as the visual-based sensors. With the proposed reference system, these sensors can also be validated after adapting the signals that are provided from the sensors. Because of the modular concept of the reference system, it is also possible to add more cameras and LiDAR sensors in order to extend the FOV of the reference system. With additional hardware and software modifications, it is possible to validate radar sensors that are placed in the corners of the vehicle, 360 degree LiDAR-based systems, or ultrasonic systems that are used for park assistance features.

These reference systems generate huge amounts of data, and the manual evaluation of the recorded data takes a lot of effort. Within this project, the development of a software solution that could automatically process and evaluate the data was started; however, the scope of the project was to create a proof of concept; the software solution is only usable with a limited amount of recorded data. There is a huge potential in developing a software solution that could process and evaluate large amount of data automatically, based on predefined conditions [37].

During a data recording campaign, it is critical to ensure the correctness of the recorded data and minimize the amount of corrupt data. For that, it is important to develop an online monitoring system that could detect any failure during the recording. The real-time visualization of the recorded data shall also be developed. With the help of the visualization, the test engineers could decide during testing whether the recorded scenario is as expected or not; therefore, it could speed up the recording and reduce the amount of unusable data. 

## Figures and Tables

**Table 1:** Measured mean and standard deviation of latency (in ms) between camera and LiDAR frames and the sensor to ROS framework latency.

Latency Type	Mean [ms]	Stdev [ms]	Remark
Camera↔LiDAR latency	3.97	0.01	
Camera↔Camera latency	0.0	0.0	HW triggered
Camera↔ROS latency	25.14	0.64	
LiDAR↔ROS latency	41.50	2.19	

**Table 2:** Comparison of different validation methods. * Communication limitation between VUT and target.

Latency Type	dGPS	UAV	Camera–LiDAR-Based System
Costs	50–100 TEUR	2–10 TEUR	120–250 TEUR
Accuracy	1–2 cm	5–30 cm	50–100 cm
Integration time	3 h	1 h	100 h
System preparation time for testing	0.5 h	0.5 h	0 h
Detection distance	400 m *	120 m	150 m
Limitations	number of targets	Flying permit	weather limitations
	urban road environment	velocity limitations	
		weather limitations	

## Data Availability

Data are contained within the article.
